# Gypenosides Attenuate Pulmonary Fibrosis by Inhibiting the AKT/mTOR/c-Myc Pathway

**DOI:** 10.3389/fphar.2021.806312

**Published:** 2022-01-14

**Authors:** Suqing Liu, Qingqing Yang, Binbin Dong, Chunhui Qi, Tao Yang, Ming Li, Shan He, Baojun Liu, Jinfeng Wu

**Affiliations:** ^1^ Department of Dermatology, Huashan Hospital, Fudan University, Shanghai, China; ^2^ Shanghai Public Health Clinical Center, Fudan University, Shanghai, China; ^3^ Department of Pediatrics, Huashan Hospital North, Fudan University, Shanghai, China; ^4^ Department of Respiratory Medicine, Qingpu District Traditional Chinese Medicine Hospital, Institute of Integrative Medicine, Fudan University, Shanghai, China; ^5^ Department of Cardiovascular Disease, Institute of Cardiovascular Disease of Integrated Traditional Chinese and Western Medicine, Shuguang Hospital Affiliated to Shanghai University of Traditional Chinese Medicine, Shanghai, China; ^6^ Department of Integrative Medicine, Huashan Hospital, Fudan University, Shanghai, China

**Keywords:** gypenosides, pulmonary fibrosis, AKT, mTOR, c-Myc

## Abstract

Gypenosides (Gyps), the major active constituents isolated from *Gynostemma pentaphyllum*, possess anti-inflammatory and antioxidant activities. Previous studies have demonstrated that Gyps displayed potent ameliorative effects on liver fibrosis and renal fibrosis. In this study, we found that Gyps significantly reduced the mortality of bleomycin-induced pulmonary fibrosis mice (40% mortality rate of mice in the model group versus 0% in the treatment group). Masson staining showed that Gyps could reduce the content of collagen in the lung tissue of pulmonary fibrosis mice Masson staining and immunohistochemistry demonstrated that the expression of the collagen gene α-SMA and fibrosis gene Col1 markedly decreased after Gyps treatment. The active mitosis of fibroblasts is one of the key processes in the pathogenesis of fibrotic diseases. RNA-seq showed that Gyps significantly inhibited mitosis and induced the G2/M phase cell cycle arrest. The mTOR/c-Myc axis plays an important role in the pathological process of pulmonary fibrosis. RNA-seq also demonstrated that Gyps inhibited the mTOR and c-Myc signaling in pulmonary fibrosis mice, which was further validated by Western blot and immunohistochemistry. AKT functions as an upstream molecule that regulates mTOR. Our western blot data showed that Gyps could suppress the activation of AKT. In conclusion, Gyps exerted anti-pulmonary fibrosis activity by inhibiting the AKT/mTOR/c-Myc pathway.

## Introduction

Pulmonary fibrosis (PF) is a chronic, progressive, and lethal interstitial lung disease, with a poor prognosis and median survival of 3–5 years after diagnosis (Masefield et al., 2019). PF is characterized by inflammation, fibroblast/myofibroblast proliferation, and the activation of alveolar epithelial cells with excessive extracellular matrix (ECM) deposition in the lung parenchyma (Wolters et al., 2014). Current pharmacological therapies include glucocorticoids, immunosuppressive drugs, and anti-fibrotic agents; however, none of them can improve the survival of patients with PF (Richeldi et al., 2017). To date, there are only two drugs approved by the U.S. Food and Drug Administration (FDA) for the treatment of PF: nintedanib and pirfenidone (Yamazaki et al., 2021). These two small molecule drugs target receptor tyrosine kinase (RTK) and the transforming growth factor (TGF-β), respectively. Although they can slow down the progression of PF, the high cost and strong toxic effects on the liver and kidneys limit their clinical application (Yang et al., 2019). In recent years, traditional Chinese medicine (TCM) has shown unique efficacies in the treatment of PF (Chen DQ. et al., 2018). Owing to the characteristics of multi-components, multi-targets, and multi-level interactions, TCM can improve the survival and life quality of the patients with PF disease to a certain extent (Wang et al., 2020).

The pathogenesis of PF is not well elucidated (Spagnolo et al., 2021). Increasing evidences showed that the progressive pulmonary scarring and lung function declining induced by the lung epithelial injury and aberrant fibroblast proliferation were involved in the pathogenesis of PF (Martinez et al., 2017). Currently, it is believed that PF originates from the repeated injury and aberrant repair of the alveolar epithelial cells (Sgalla et al., 2018). Repeatedly injured alveolar epithelial cells secrete PF-related growth factors, such as the connective tissue growth factor (CTGF), transforming growth factor β1 (TGF-β1), and insulin-like growth factors (IGF-1), which activate the phosphatidylinositol 3-kinase (PI3K)/AKT signaling pathway through binding to the corresponding receptors on the lung fibroblast membrane, to promote lung fibroblast proliferation and collagen synthesis and induce the epithelial-to-mesenchymal transition (EMT) (Phan et al., 2021). As a downstream target of the PI3K/AKT signaling pathway, excessive activation of mTOR in the alveolar epithelium exacerbates bleomycin (BLM)-induced PF in mice (Cong et al., 2020). Moreover, c-Myc, a star transcription factor in the downstream of mTOR, can stimulate fibroblast proliferation in the presence of growth factors (Lawrence and Nho, 2018).

Gypenosides (Gyps), the saponin extracts of *Gynostemma pentaphyllum*, have been extensively studied in fibrosis diseases, such as liver fibrosis and renal fibrosis, for their anti-inflammatory, anti-fibrotic, antioxidant, and anti-apoptotic effects (Nguyen et al., 2021). In this study, we evaluated that Gyps attenuated BLM-induced PF in mice through mediating the AKT/mTOR/c-Myc pathway.

## Materials and Methods

### Instrument and Chromatographic Conditions

Chromatography was performed using a Dionex Ultimate 3000 ultra-high-performance liquid chromatography (HPLC) system from Thermo Fisher Scientific (Waltham, MA, United States) and an ACQUITY UPLC HSS T3 column (2.1 mm × 100 mm, 1.8 μm, Waters Corp., Milford, MA, United States). The components were eluted with a gradient elution system consisting of 0.1% formic acid aqueous solution (A) and acetonitrile (B). The mobile phase gradient elution was programmed as follows: 0–12 min, 5% B–95% B; 12–14 min, 95% B; 14.01 min, 5% B; and 14.01–16 min, 5% B. The mobile phase flow rate was 0.3 ml/min, and the column temperature was maintained at 45°C.

Mass spectrometric detection was performed using a quadrupole mass spectrometer equipped with electrospray ionization (HESI) in the positive and negative mode under the following operating parameters: the ion source was operated with a capillary voltage set to 3.5 kV (ESI+) and 2.8 kV (ESI-), the capillary temperature at 320 °C, the auxiliary gas heating temperature at 35°C, sheath gas (nitrogen) flow at 80 AU, and auxiliary gas (nitrogen) flow at 13 AU, respectively. Data acquisition was performed full-scan, selective ion monitoring (SIM) mode in the range of m/z 80–1,200.

### Preparation of Standard Solutions and Sample Solutions

The mixture of a standard stock solution containing above eight compounds (ginsenoside Rb1, ginsenoside Rd, rutin, quercetin, kaempferol, gypenoside XLIX, ombuoside, and ombuin) was prepared in methanol at 1 μg/ml. Furthermore, the chemical constituents of the total saponin extract of *Gynostemma pentaphyllum* (Gypenosides, Gyps) was extracted by ultrasonicating in methanol for 30 min at 250 W and 40 kHz. Then, 100 μL aliquot of the sample solution which was filtered by a 0.22 μm millipore filter was injected into the chromatographic systems for analysis.

### Reagents

Primary antibodies against mice α-SMA, Col1, c-Myc, INSC, AKT, p-AKT, mTOR, p-mTOR, and β-actin were purchased from Abcam (Cambridge, United Kingdom), and secondary antibodies were obtained from Yeasen Biotech Co., Ltd. (Shanghai, China). Gyps were bought from Ronghe Co., Ltd. (Shanghai, China). BLM was supplied by MCE (Shanghai, China).

### Animal Experimental Procedure

Six-week-old female C57BL/6 J mice were purchased from Vital River Laboratory Animal Technology Co. (Beijing, China). All mice were housed in a pathogen-free facility at the animal room of the Shanghai Public Health Clinical Center. The mice were raised at proper humidity (60 ± 2%) and temperature (25 ± 2°C) and allowed free access to food and water. All animal experimental procedures were reviewed and approved by the animal ethics committee of the Shanghai Public Health Clinical Center (permit number: 2020-A037-01). Twenty-five mice were assigned to three groups at random: the control group (5 mice), BLM group (10 mice), and Gyps-treated group (10 mice). For BLM and Gyps-treated group, mice were anesthetized and then intratracheally injected with BLM at a dose of 3 mg/kg to induce the fibrotic response at day 0. The control group received intratracheal injection of the same amount of saline. The Gyps were administered daily from day 1 to day 20 by gavage at a dose of 200 mg/kg in the Gyps-treated group. The BLM group was given the same amount of saline by gavage. Mice were sacrificed on day 21. Briefly, the heart was perfused with PBS through the right ventricle until the lung was clear of blood after anesthetized. The right lung tissue was isolated for Western blot assay and RNA-sequencing, and the left lung tissue was harvested for histology assay.

### Histology and Immunohistochemistry

Histology and immunohistochemistry assays were performed and analyzed, as described in our previous article (Li Q. et al., 2020). Briefly, after being fixed with 4% formaldehyde, the left lung was paraffin-embedded and sliced into 4–6 um thick sections. Deparaffinized sections were stained with hematoxylin and eosin (HE) and followed by staining with Masson’s trichrome and periodic acid–Schiff (PAS). Immunohistochemical analysis was performed on 4-μm formalin-fixed sections using the primary antibody against α-SMA, Col1, and c-Myc.

### Western Blot

Total protein was extracted from lung tissue homogenates using the RIPA reagent (Beyotime Biotechnology, lnc, JiangSu, China) supplemented with 1% PMSF (Beyotime) and separated through electrophoresing on 10% SDS-PAGE gels (30μg/lane). The separated protein was transferred to the polyvinylidene difluoride (PVDF) membranes (Merck Millipore, lnc., Darmstadt, Germany) and subsequently blocked with 5% skimmed milk. The targeted protein was probed with antibodies against INSC, AKT, p-AKT, mTOR, p-mTOR, and β-actin (all from Abcam, Cambridge, United Kingdom). After an incubation step with horseradish peroxidase (HRP)-conjugated secondary antibodies, the bands were visualized using an enhanced chemiluminescence kit (Merck Millipore).

### RNA Sequencing Analysis

Total RNA of the lung tissue was isolated using the miRNeasy Micro Kit (Qiagen, Hilden, Germany), and the concentration and purity of RNA were detected by using a Bioanalyzer 4200 (Agilent, Santa Clara, CA, United States). RNA-seq analysis was performed, as described in our previous article (Li H. et al., 2020).

### Statistical Analysis

Statistical analysis was performed by GraphPad Prism 8 software (GraphPad Software, Inc., San Diego, CA). Data were analyzed by student’s t-test or one-way *ANOVA* which were followed by Turkey’s *post hoc* analysis for the comparison of two or more independent groups, respectively. Survival data were analyzed using Kaplan–Meier survival analysis. A *p* value less than 0.05 was regarded as a statistically significant difference.

## Results

### UPLC Chromatograms of Gyps

**FIGURE 1:**
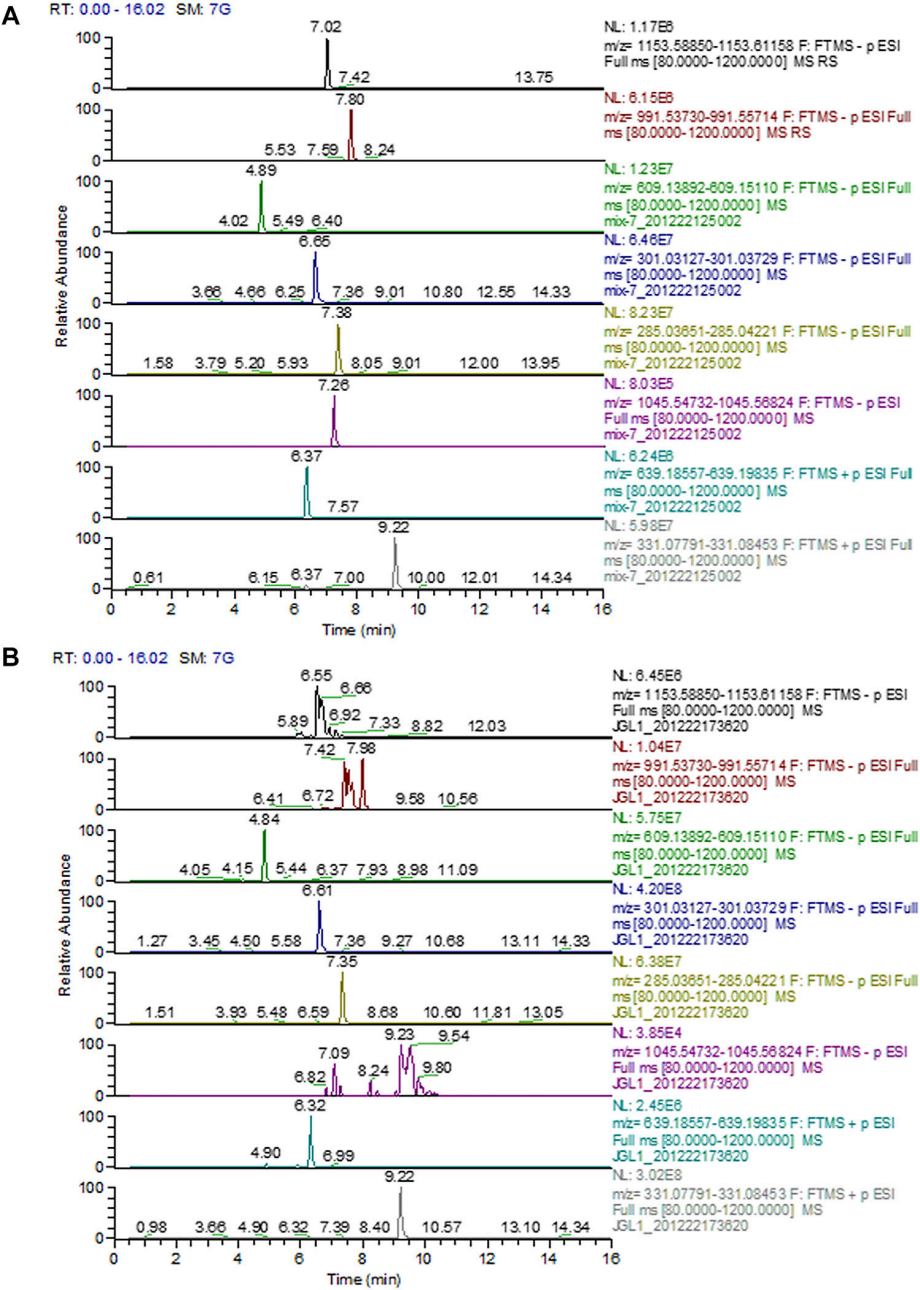
Representative full-scan chromatograms of **(A)** Standard solutions; **(B)** Sample solution: ginsenoside Rb1 (6.92 min), ginsenoside Rd (7.98 min), rutin (4.84 min), quercetin (6.61 min), kaempferol (7.35 min), gypenoside XLIX (7.26 min), ombuoside (6.32 min), and ombuin (9.22 min).

The chromatograms of mixed standards and Gyps were illustrated in Figure 1. The contents of ginsenoside Rb1, ginsenoside Rd, rutin, quercetin, kaempferol, gypenoside XLIX, ombuoside, and ombuin in Gyps were approximately detected to be 0.01713, 0.02351, 0.07513, 0.09762, 0.01106, 0.00017, 0.05242, and 0.08317%, respectively.

### Gyps Enhanced the Survival Rate of BLM-Induced PF Mice

**FIGURE 2:**
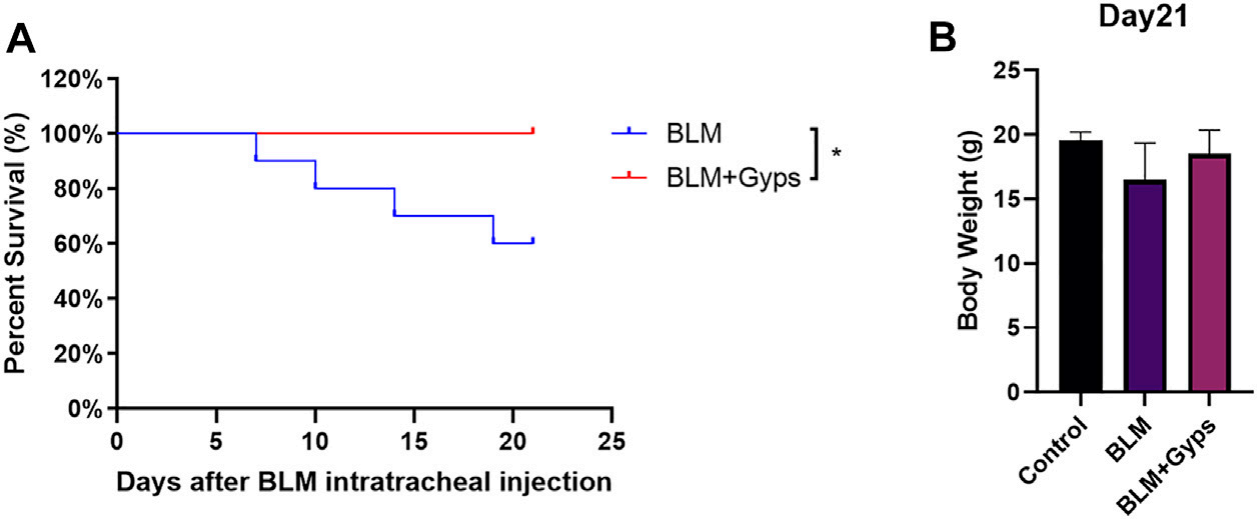
Effects of Gyps on PF development induced by BLM in mice. **(A)** Survival curves of mice in each group. (*n* = 10). The PF mouse model was established on day 0. **(B)** Mouse body weights were measured on day 21. Gyps = gypenosides, BLM = bleomycin.

As shown in Figure 2A, the death of BLM-induced PF mice occurred from day 7 after BLM intratracheal injection, and Kaplan–Meier survival curves demonstrated that BLM-induced PF mice treated with Gyps had a significantly higher survival rate than those treated with saline (100 vs. 60% 21-days survival, log-rank test, *p* < 0.05, Figure 2A). However, there was no significant difference in the body weight among the three groups on day 21 (Figure 2B).

### Gyps Ameliorated Pulmonary Inflammation and Fibrosis

**FIGURE 3:**
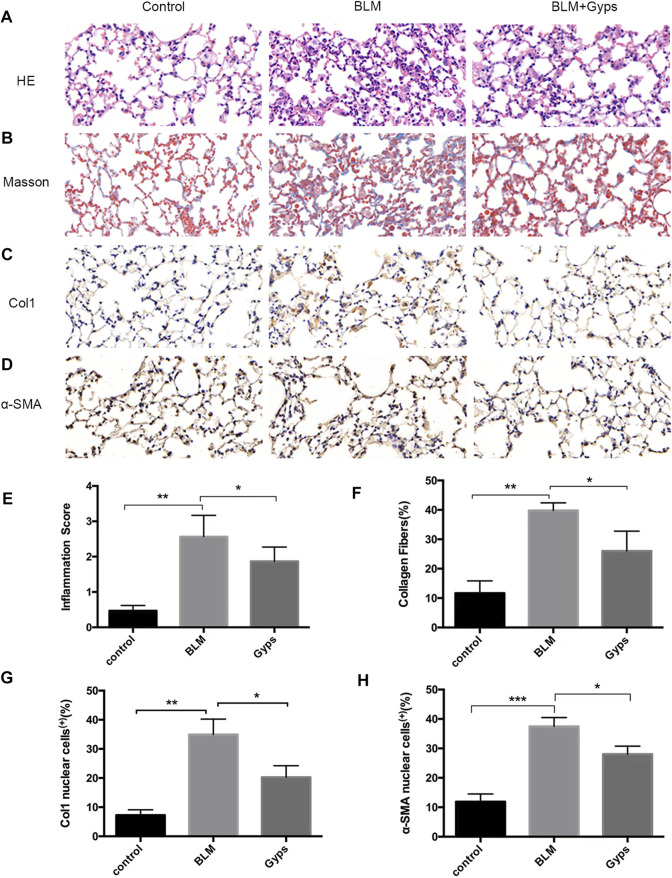
Effects of Gyps on pulmonary inflammation and fibrillation. **(A, B)** Representative images of HE and Masson’s staining of lung tissue sections (400×). **(C, D)** Immunohistochemistry was used to analyze the expression levels of Col1 and α-SMA (400×). **(E–H)** Quantitation of the data presented in panel **(A–D)**. Gyps = gypenosides, BLM = bleomycin. ***p < 0.001, **p < 0.01, compared with control; *p < 0.05 compared with BLM.

The normal alveolar structure was maintained in the control group (Figure 3A). Twenty-one days after BLM injection, HE and Masson’s trichrome staining showed a remarkably thick alveolar wall and collapse of alveolar septa, inflammatory cell infiltration, loss of lung architecture, and excess deposition of collagen in the BLM-treated group compared with the control group, whereas treatment with Gyps markedly attenuated the injury of lung architecture and the deposition of collagen caused by BLM (Figures 3A,B,E,F). The effects of Gyps on the BLM-induced collagen deposition and alveolar fibrosis in the lungs were further investigated by immunohistochemistry assay. The data showed that BLM upregulated the expressions of the collagen gene α-SMA and fibrosis gene Col1 in the lung tissue compared with the control group, while the expressions of α-SMA and Col1 in the lung tissue of Gyps-treated mice were significantly lower than those in the model mice (Figures 3C,D,G,H).

### Gyps Alleviated PF by Inhibiting Mitosis

**FIGURE 4:**
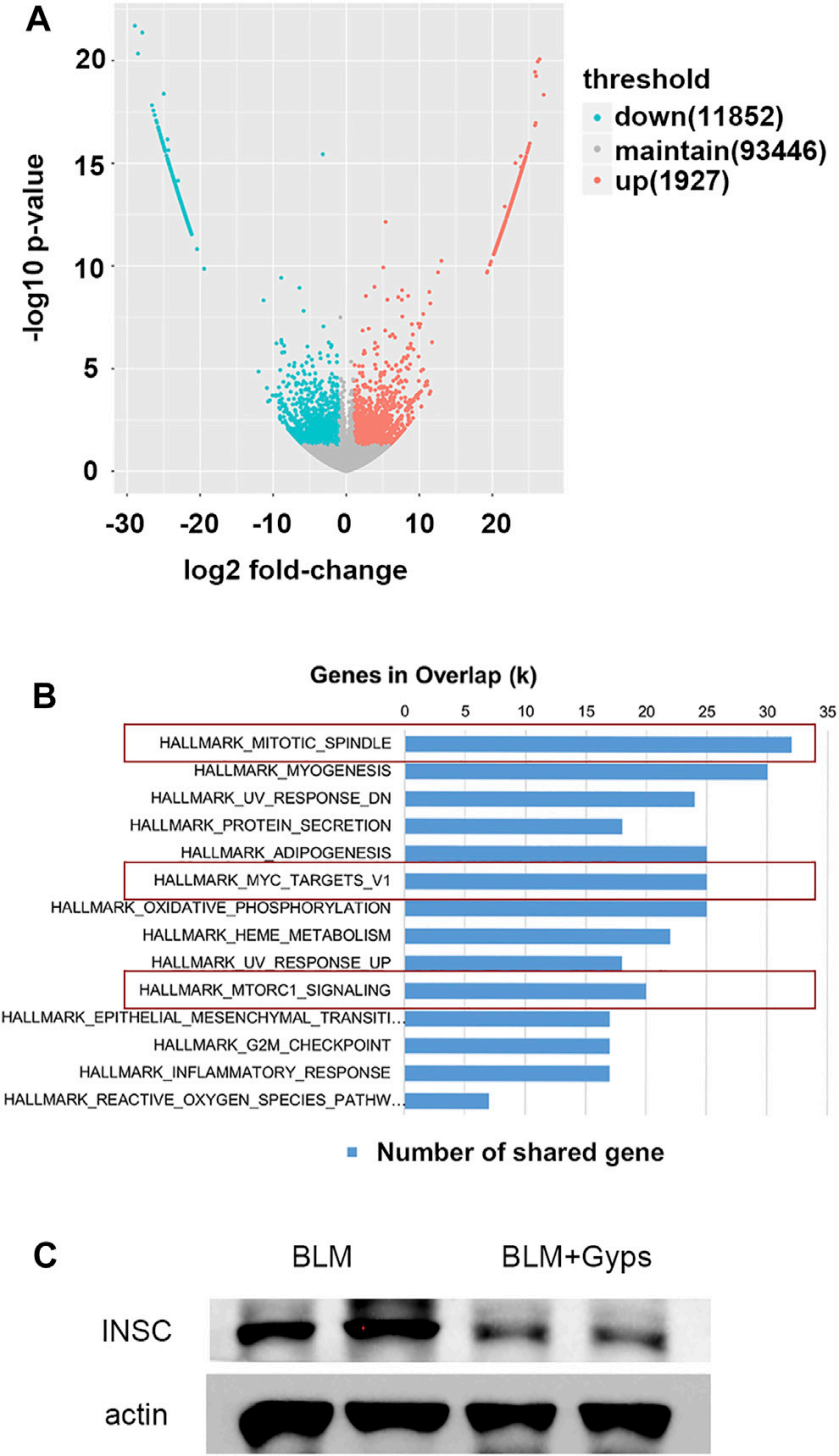
RNA-seq analysis and Hallmark analysis. **(A)** RNA-seq analysis was performed, and the volcano plot was demonstrated. (n = 5 mice/group) **(B)** Results of Hallmark analysis on downregulated transcripts in the Gyps group compared with that in the control group. **(C)** Protein expression levels of INSC as detected by Western blotting. Gyps = gypenosides, BLM = bleomycin.

We then performed RNA-seq of the RNA isolated from the lung tissues to further explore the possible mechanisms. As demonstrated in Figure 4A, 11,852 downregulated transcripts and 1927 upregulated transcripts were found in the Gyps-treated group compared with the BLM group (*n* = 5 mice/group). Gene set enrichment analysis of the downregulated transcripts enriched in the Hallmark gene sets (Figure 4B) illustrated that mitotic spindle-related transcripts (top 1 ranked) were significantly downregulated in the Gyps-treated group in comparison with the BLM group. It has been reported that the active mitosis of fibroblasts was one of the key processes in the pathogenesis of fibrotic diseases (Adamson, 1984; Tomcik et al., 2016). INSC functions as an adapter protein that was involved in spindle orientation during mitosis (Culurgioni and Mapelli, 2013). As shown in Figure 4C, Gyps treatment significantly decreases the expressions of INSC, which was highly expressed in the BLM group. We also found that Gyps induced the G2/M cell cycle arrest (ranked 12) (Figure 4C).

### Gyps Inhibited Mitosis by Repressing the mTOR/c-Myc Axis in PF Mice

**FIGURE 5:**
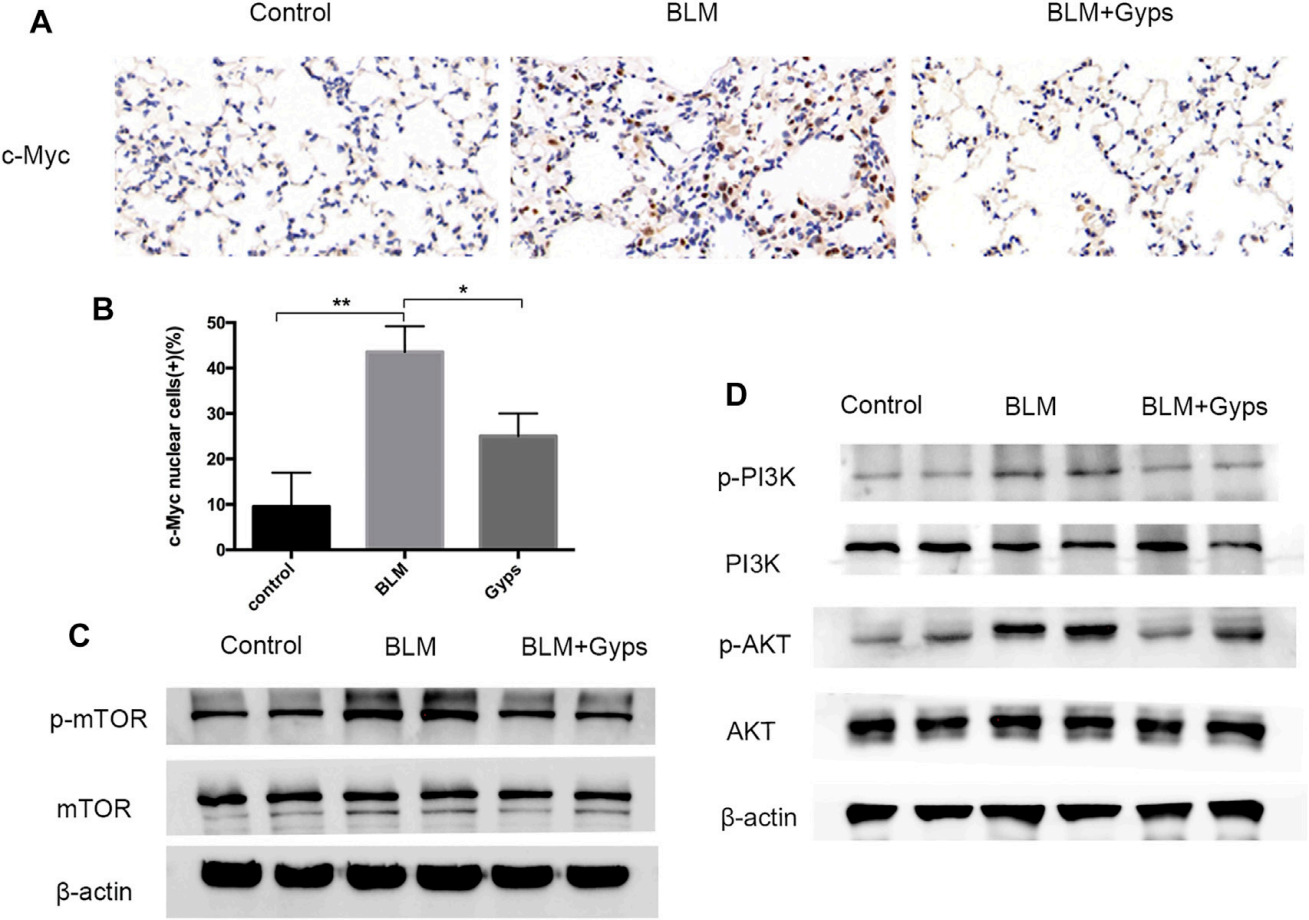
Expressions of c-Myc, p-mTOR, mTOR, p-PI3K, PI3K, p-AKT, and AKT in the mouse model. **(A)** c-Myc Immunohistochemical staining (400×). **(B)** Quantitation of the data presented in panel **(A)**. **(C, D)** Protein expression levels of p-mTOR, mTOR, p-PI3K, PI3K, p-AKT, and AKT as detected by Western blotting. Gyps = gypenosides, BLM = bleomycin. ***p < 0.001, **p < 0.01, compared with control; *p < 0.05 compared with BLM.

As demonstrated in Figure 4B, the Hallmark analysis also indicated that Myc (ranked 6) and mTORC1 (ranked 10) signaling-related genes were notably inhibited after Gyps treatment. Myc family has a central role in orchestrating cell proliferation (Chen H. et al., 2018). The mTORC1 controls cell growth and metabolism in response to nutrients, energy levels, and growth factors (Yang et al., 2017). c-Myc is one of the key target genes of mTOR, and mTOR and c-Myc form an axis. They are highly related to the mitotic process (Cianfanelli et al., 2015). Our IHC analysis showed that Gyps inhibited the expression of c-Myc in BLM-injected mice (Figures 5A,B). Meanwhile, we found that Gyps administration obviously inhibited the BLM-induced increase of p-mTOR by Western blot assay (Figure 5C).

### Gyps Inhibited the mTOR/c-Myc Axis by Regulating PI3K/AKT Signaling

PI3K/AKT signaling is one of the key cellular signaling pathways which regulate cell proliferation, growth, metabolism, and motility. mTOR and c-Myc are the downstream proteins of PI3K/AKT signaling (Alzahrani, 2019). Thus, we detected the expressions of p-PI3K, PI3K, p-AKT, and AKT in the lung tissues and found (Figure 5D) that the expression of p-PI3K and p-AKT in BLM-induced PF mice showed a significant increase compared with that of the control group. Interestingly, the expressions of p-PI3K and p-AKT were markedly reduced after treating with Gyps, which demonstrated that Gyps could suppress the mTOR/c-Myc axis by inactivating PI3K/AKT signaling.

## Discussion

Gyps, the total saponins extracted from *Gynostemma pentaphyllum*, were widely used for anti-inflammation, antioxidant, antitumor, and immune enhancement. The anti-fibrotic effects of Gyps have been reported in multiple fibrotic diseases including liver fibrosis, renal fibrosis, and Graves’ ophthalmopathy (Chen et al., 2017; Li H. et al., 2020; Liu Q. et al., 2021). In this study, we first reported that Gyps could alleviate BLM-induced pulmonary fibrosis in mice. We found eight major components in Gyps, among which ginsenoside Rb1, rutin, quercetin, kaempferol, and gypenoside XLIX have been reported to have anti-fibrotic effects (Hou et al., 2014; Verma et al., 2017; Wu et al., 2017; Avila-Carrasco et al., 2019; Liu et al., 2019; Bai et al., 2020; Liu et al., 2020; Liu H. et al., 2021). Based on our *in vivo* data, 40% of PF mice died, while none of the Gyps treated PF mice died. This indicated that Gyps could significantly improve the survival rate of PF mice and had a good promise for the treatment of pulmonary fibrosis. However, administration of Gyps could not attenuate the effects of BLM on body weight loss in PF mice. It might be because most of the low-weight PF mice died.

**FIGURE 6:**
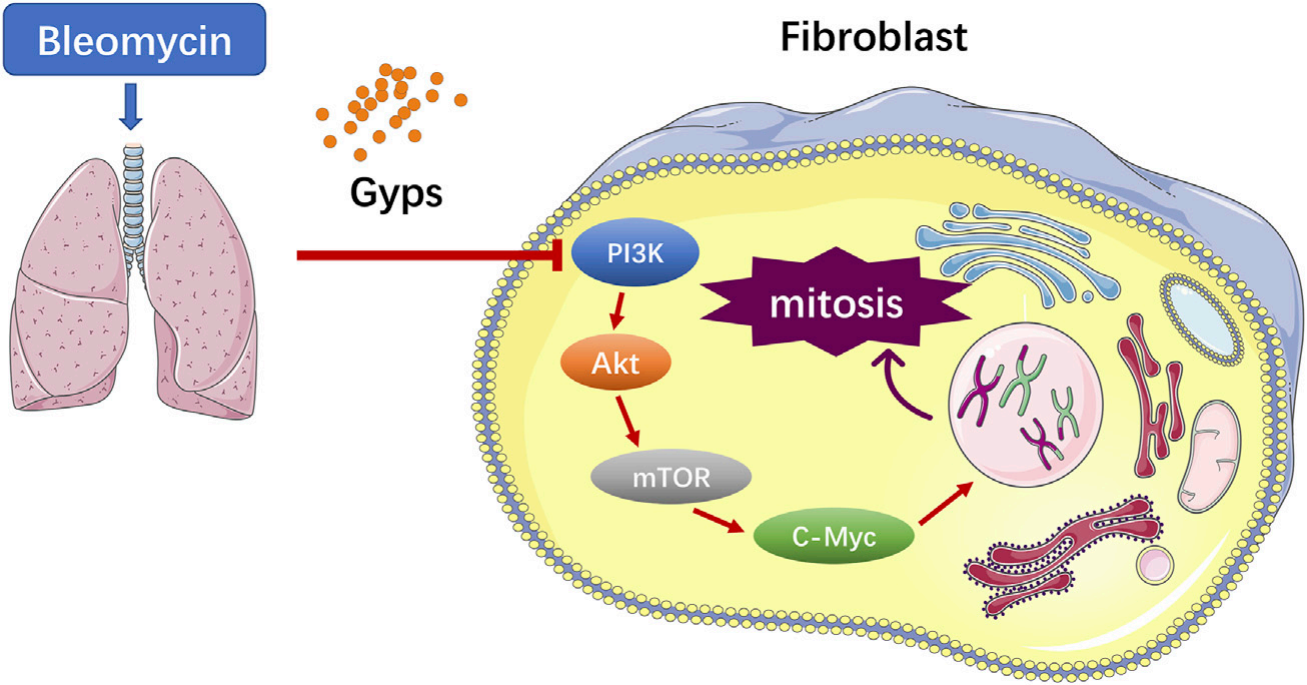
Potential mechanism of Gyps on PF mice induced by BLM. Gyps = gypenosides.

PF is characterized by increased fibroblast proliferation and ECM protein deposition by myofibroblasts under the control of pro-fibrogenic stimuli such as TGF-β1 (Davies et al., 2012). Based on the analysis of RNA-seq, the Gyps-treated group showed the most significant improvement in mitosis and promoted G2/M cell cycle arrest. In the future, the *in vitro* experiment should be carried out to verify if Gyps could directly inhibit mitosis in pulmonary fibroblasts. It has been reported that some non–SMAD-mediated pathways including PI3K/AKT, RhoA, PAR6, and MAPK pathways could lead to EMT in the fibrotic process (Cho et al., 2007; Willis and Borok, 2007). PI3K is an intracellular phosphatidylinositol kinase, which has serine/threonine kinase and phosphatidylinositol kinase activities (Yang et al., 2020). AKT is a serine/threonine kinase and is directly activated in response to PI3K which functions as an important regulator of cell growth, survival, and glucose metabolism (Barrett et al., 2012). Yuan et al. reported that miR-410 induces EMT through activating the PI3K/mTOR pathway in non–small cell lung cancer (Yuan et al., 2020). Recent studies have shown that the phosphorylation of AKT was increased in human pulmonary fibroblasts induced by BLM and was also increased in radiation-induced PF (Ma et al., 2020a; Ma et al., 2020b). The atypical serine/threonine kinase mTOR is the key downstream target molecule of AKT and functions as an important regulator of cell growth, metabolism, and immunity (Hua et al., 2019). c-Myc, a star transcription factor in the downstream of mTOR, can stimulate fibroblast proliferation in the presence of growth factors (Lawrence and Nho, 2018). Decreased c-Myc expression mediated fibronectin and collagen deposition into the extracellular matrix and attenuated cell proliferation in the fibrosis process (Valiente-Alandi et al., 2018). Shin et al. revealed that the AKT inhibitor LY294002 blocked the expressions of p-AKT and c-Myc in HepG2 cells and found that the compound K induced apoptosis in hepatocellular carcinoma cells *via* inhibition of AKT/mTOR/c-Myc signaling (Shin et al., 2021). Liu et al. reported Gyps-induced apoptosis of renal cell carcinoma cells through regulating the PI3K/AKT/mTOR signaling pathway. (Liu H. et al., 2021). In the present study, we demonstrated that Gyps markedly inhibited the AKT/mTOR/c-Myc pathway in pulmonary fibrosis (Figure 6).

Many growth factors are upstream regulators of PI3K/AKT signaling, such as CTGF, TGF-β1, and IGF-1, which are reported to be involved in the pathological process of PF. Gyps were reported to protect orbital fibroblasts in Graves’ ophthalmopathy *via* downregulating TGF-β–induced fibrotic mediators (Li H. et al., 2020). In another study, researchers reported that Gyps ameliorated CCl_4_-induced liver fibrosis *via* inhibiting TGF-β1 signaling and consequently inhibiting the differentiation of hepatic progenitor cells into myofibroblasts (Chen et al., 2017). In a unilateral ureteral obstruction-induced tubulointerstitial damage and fibrosis model, the expressions of TGF-β1 and CTGF were significantly reduced by Gyps treatment, and the Smad7 expression was elevated by Gyps treatment (Li Q. et al., 2020). In the future, we will determine if Gyps could affect the upstream regulators of PI3K/AKT signaling in PF.

EMT, inflammatory response, and reactive oxygen species (ROS) pathways are involved in the pathological process of PF (Wolters et al., 2014). EMT is a process in which epithelial cells gradually acquire a mesenchymal (fibroblast-like) cell phenotype (Bartis et al., 2014). A previous study reported that in a repetitive bleomycin injury PF model, about 50% of S100A4+ fibroblasts were epithelial-derived using genetic fate tracking (Johnson et al., 2011). It was reported that polyhexamethylene guanidine-phosphate infiltrated into the lungs in the form of aerosol particles would induce an airway barrier injury by generating ROS, releasing fibrotic inflammatory cytokines, and triggering a wound-healing response, thus leading to pulmonary fibrosis (Kim et al., 2016). Our RNA-seq data suggested that Gyps had anti-inflammatory, antioxidative, and anti-EMT effects (ranked 11, 13, and 14, respectively) in PF mice. Further studies are needed to confirm the above effects of Gyps in PF.

In conclusion, we revealed that Gyps attenuated PF development in mice, and the potential mechanism was due to inhibiting the AKT/mTOR/c-Myc axis. Gyps might be a promising candidate drug for the treatment of pulmonary fibrosis.

## Data Availability

The datasets presented in this study can be found in online repositories. The names of the repository/repositories and accession number(s) can be found in the article/Supplementary Material.
